# The Complex Etiology of Epilepsy: Genetic Analysis and HLA Association in Patients in the Middle East

**DOI:** 10.3390/ijms26125815

**Published:** 2025-06-17

**Authors:** Abeer Fadda, Mohamed Alsabbagh, Dhanya Vasudeva, Amira Saeed, Sara Aglan Tarek, Satanay Z. Hubrack, Ruba Benini, Khaled Zamel, Bernice Lo

**Affiliations:** 1 Research Department, Sidra Medicine, Doha 26999, Qatar; malsabbagh@sidra.org (M.A.); sara.aglantarek@gmail.com (S.A.T.); shubrack@sidra.org (S.Z.H.); blo@sidra.org (B.L.); 2 Division of Pediatric Neurology, Department of Pediatrics, Sidra Medicine, Doha 26999, Qatar; dvasudeva@sidra.org (D.V.); asaeed@sidra.org (A.S.); rbenini@sidra.org (R.B.); 3 Department of Clinical Pediatrics, Weill-Cornell Medical College, Doha 24144, Qatar; 4 College of Health and Life Sciences, Hamad Bin Khalifa University, Doha 34110, Qatar

**Keywords:** epilepsy, HLA, genetics, immunology

## Abstract

Epilepsy is one of the most common neurological disorders. Disease etiology and pathogenesis are still not well understood. Genetic mutations are associated with 70% of epilepsies, while 30% are still enigmatic. Attempting to close the knowledge gap, we performed genetic analysis of a cohort of patients from the Middle East and North Africa, both understudied and highly consanguineous populations. Whole exome sequencing (WES) was carried out on 81 patients and their family members at a tertiary center in Qatar. We found damaging mutations in half of the patients: 15 in known epilepsy genes, and 19 in contested or unknown genes. The mutations include single nucleotide polymorphisms (SNVs), frameshifts, copy number variations (CNVs), and loss of homozygosity (LOH). Fifteen of the SNVs are novel, and seventeen are homozygous, reflective of the characteristics of the cohort. In addition, we used the WES data to type HLA alleles for 13 class I and II genes. We show that DRB3*01:01:02G is negatively associated with epilepsy, in contrast to DRB4*01:01:01G, which may be a risk allele. In addition to expanding the knowledge base of genes involved in epilepsy, our findings show that genetic predisposition, inclusive of immune genes, suggests a complex etiology.

## 1. Introduction

Epilepsy is a chronic neurological disorder characterized by an increased predisposition for epileptic seizures. Genetic abnormalities reportedly cause or influence more than 70% of epileptic conditions [1]. Even after a thorough diagnostic evaluation, a large proportion of patients have epilepsy of an unknown etiology [2]. However, 4% to 78% of selected patients with initially unknown etiology have genetic variants of probable or definitive etiologic significance [3]. The wide range in the predicted proportions of patients with a genetic contribution to epilepsy reflects the complex and heterogeneous nature of the disorder, as well as the ongoing efforts to understand the underlying genetic and environmental causes.

The identification of epilepsy genes originated from the analysis of deleterious variants in affected individuals. Presently, databases such as OMIM list around 177 epilepsy genes, Genomics England panel includes 575 validated genes, and commercial testing panels (e.g., Invitae) encompass approximately 300 genes. The phenotypic diversity and pleiotropy of these genes challenge the definition of a specific set of epilepsy genes. As a result, various inheritance models are proposed, ranging from full-penetrant monogenic to additive polygenic, where each involved gene contributes as a risk factor. Several big initiatives were launched to discover and assess the contribution of genes in epileptogenesis, such as the Epi25K consortium, the Epilepsy Phenome/Genome Project, Epicure, EuroEPINOMICS, CoGIE, EpiPGX, and ILAE Consortium. With thousands of samples, these consortia were able to use non-Mendelian analysis methods: genome-wide association studies (GWAS), genetic burden, or risk scores, enabling the discovery of more risk genes.

Beyond genetic origins, the role of the immune system, though recognized, remains incompletely understood in epilepsy. The latest ILAE classification designates autoimmune epilepsies as a distinct group. Evidence suggests neuroinflammation as both a trigger for epileptogenesis and a response to epileptic activity [4,5,6] and associations with variants in immune genes, including HLA, have been identified [7,8]. Challenges in obtaining brain tissue constrain our comprehension of the immune system’s extent of involvement in diverse epilepsy types.

To address gaps in epilepsy etiology, we utilized a MENA region cohort, traditionally underrepresented but enriched with consanguinity. Employing whole exome sequencing on a cohort of 67 pediatric epilepsy patients and their familial counterparts, we interrogated for pathogenic variants. Our analysis revealed an approximate yield of 30% for known epilepsy-associated genes. In silico HLA typing revealed positive associations with epilepsy for DRB1*07:01:01G and DRB4*01:01:01G group alleles, with DRB3*01:01:02G showing a negative association. The presence of HLA risk alleles in genetically clear cases suggests a potential overlap between genetic and immune epilepsies, challenging previous distinctions.

## 2. Results

### 2.1. Cohort Demographics and Phenotype

Our cohort consisted of 67 families followed in the pediatric neurology department at Sidra Medicine, the only tertiary pediatric hospital in Qatar. Recurrent unprovoked seizures of unknown etiology, and an epilepsy diagnosis based on clinical and EEG data, were among the inclusion criteria. We excluded patients with an identified antecedent cause of epilepsy (i.e., a structural or metabolic insult to the central nervous system prior to the first unprovoked seizure such as stroke, brain tumor, severe head trauma, etc., or a progressive neurodegenerative disorder). We also excluded patients with recognized genetic syndromes or chromosomal abnormalities that explained the epilepsy. Neuroimaging findings that were typical of autoimmune epilepsies were not used as an exclusion criterion.

Parents and available affected or unaffected siblings were recruited to the study upon consent in accordance with the internal review board guidelines. Data collected included age of the patients at the last clinic visit, nationality, family history including presence of consanguinity, antenatal history, previous medical history, and other associated medical history. Details of the epilepsy history included the age at onset of seizures, semiology, antiepileptic medications, EEG findings as well as whether other treatment modalities were used such as dietary therapies, vagal nerve stimulation, or epilepsy surgeries. The seizure and epilepsy types were determined as per the 2017 ILAE classification [9].

**Figure 1:**
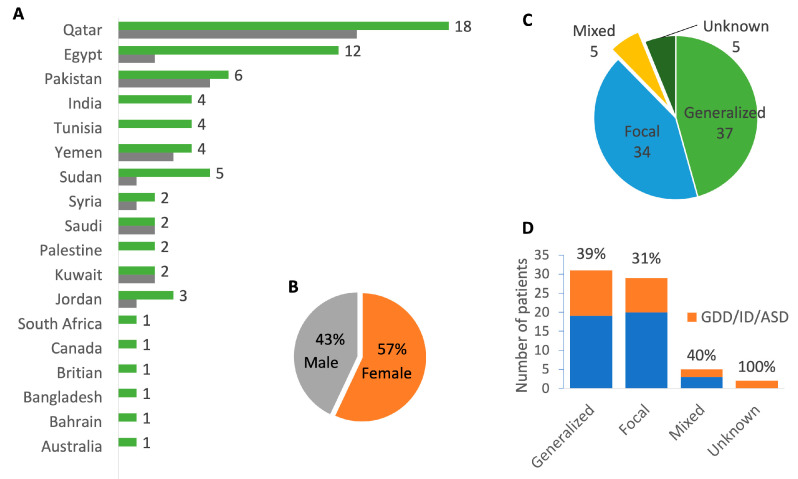
Overview of patients’ demographics and phenotypes. (**A**) Total number of families by nationality (green bars) and number of consanguineous families (gray bars). (**B**) Sex distribution. (**C**) Distribution of epilepsy classes by number of patients. (**D**) Cumulative bar plot showing the ratio of patients with GDD/ID/ASD in each epilepsy class, with percentage displayed on top.

We performed WES on a total of 225 samples for 67 families: one as proband only, five as duo, 48 as trio, and 13 as quad+ (see pedigrees in Supplementary Figure S2). The cohort included 81 affected subjects of varied ages, with the youngest being 3 weeks old. The majority were from the MENA region, and 11 were from the Indian subcontinent (Figure 1). A total of 33% of the families were formed of consanguineous marriages between 1st and 2nd cousins. A total of 40% of patients had either global developmental delay (GDD) and/or intellectual disability (ID) or autism spectrum disorder (ASD) (Figure 1). Demographic and phenotypic details, including seizure type, age of seizure offset, EEG findings, intractability, epilepsy diagnosis, comorbidities, and family history are presented in Supplementary Table S1 (clinical data sheet).

### 2.2. Identification of Deleterious Variants in High Confidence Genes

The Genomics England panel for “genetic epilepsy syndrome” [10] (Version 4.0) was used as a benchmark for known epilepsy genes. Genes in the panel have a level-of-confidence color code: green being of diagnostic grade, while amber and red indicate moderate and low levels of evidence, respectively. We filtered variants revealed by WES of our cohort, for damaging variants in “green” genes, as described in the methods. This analysis resulted in a yield of 15 families with at least one affected epilepsy gene. The affected genes are: ALKBH8, ATP1A2, CACNA1C, CACNB4, CDKL5, HECW2, HSD17B4, KCTD3, PCDH19, PRRT2, RELN, RNASEH2B, SARS1, SCN1A, SPTAN1. The variants consisted of 11 SNVs, two frameshifts, two CNVs, and two splice site deletions. Nine of the variants are novel with no assigned ID in dbSNP (Supplementary Table S1, Genetic data sheet). While most variants follow an autosomal dominant inheritance pattern, we highlight four instances of autosomal recessive inheritance, three of which harbored in patients of consanguineous families. Seven of the variants in the “green” genes were confirmed by independent clinical genetic testing (underlined in Supplementary Table S1). A brief description of each patient and the deleterious variants they harbor is provided below, and additional details are included in Supplementary Table S1.

EPBL-0010 presented with a complex phenotype: generalized epilepsy, GDD, and autism. The mother is affected with epilepsy and the older sister with paroxysmal kinesigenic dyskinesia. A pathogenic frameshift variant in PRRT2 (Arg217ProfsTer8) was found in all three affected family members (the genetic analysis for the sister was performed elsewhere). In addition, we detected a de novo variant in SCN9A (p.Tyr63Cys), classified as a variant of uncertain significance (VUS), which was present only in the proband. Although SCN9A is currently listed as a “red” gene on the epilepsy PanelApp due to limited evidence supporting its role as a monogenic epilepsy gene [11], it may act as a susceptibility factor. Considering this, the SCN9A variant may contribute to the more severe clinical presentation observed in the proband compared to other affected family members.

Patient EPBL-0013 suffers from epileptic encephalopathy and ID, and his unaffected parents are first cousins. We found two damaging heterozygous variants in ATP1A2 (Trp928Ter) (pathogenic) and HECW2 (Asn1199Lys) (likely pathogenic). Since the parents declined to be included in the study, we cannot confirm the mode of inheritance.

EPBL-0016 with Dravet syndrome has a large de novo deletion encompassing several sodium channel coding genes including the well-known epilepsy gene SCN1A.

**Figure 2:**
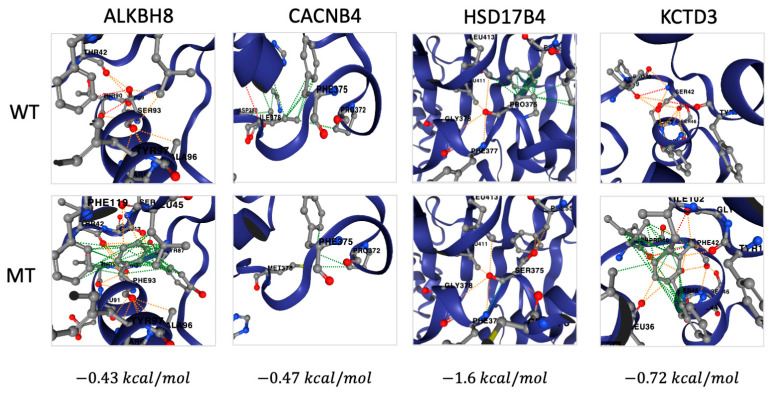
Examples of predicted destabilizing effects of variants on protein structure. Wild type (WT) and mutated (MT) protein structures predicted by AlphaFold and DynaMut2 for ALKBH8, CACNB4, HSD17B4, and KCTD3 genes. Numerous changes in intermolecular hydrophobic (green) and polar (red) bonds are shown, with the destabilizing effects quantified by the ΔΔG^Stability^ value displayed at the bottom.

A homozygous deleterious variant in ALKBH8 (Ser96Phe) (VUS) was found in EPBL-0022, a 10 year old female who presented with generalized epilepsy and GDD and diagnosed with Lennox–Gastaut syndrome. The variant is predicted to introduce multiple hydrophobic interactions, destabilizing the protein, with a ΔΔG^Stability^ = −0.43 kcal/mol (Figure 2).

EPBL-0064 presented with generalized epilepsy, ID, and ADHD. A de novo splice site deletion was found in RNASEH2B exon 6 (c.437-3_437delAAGG) (likely pathogenic). While damaging biallelic variants in RNASEH2B often result in Aicardi–Goutières Syndrome (AGS), our patient lacks such biallelic variants and does not exhibit AGS symptoms.

A homozygous deleterious variant was found in HSD17B4 (Pro375Ser) (VUS) in both patient EPBL-0073 and her younger sister (EPBL-0076) of a consanguineous family. The sisters suffer from focal epilepsy, hypotonia, and GDD. The younger sister passed away of cardiac arrest at the age of 5 months. The mutation is predicted pathogenic by AlphaMissense (Supplementary Table S1) and predicted by DynaMut2 to eliminate several intermolecular hydrogen bonds, destabilizing the protein with a ΔΔG^Stability^ = −1.6 kcal/mol (Figure 2).

A homozygous deleterious variant was found in KCTD3 (Ser42Phe) (VUS) in two siblings of a consanguineous family (EPBL-0077 and -0078). The sister and brother presented with focal epilepsy and no other co-morbidities. The mutation is predicted to be pathogenic by AlphaMissense (Supplementary Table S1) and predicted by DynaMut2 to introduce many intermolecular hydrogen bonds, resulting in protein destabilization with a ΔΔG^Stability^ = −0.72 kcal/mol (Figure 2).

An X-linked maternally inherited heterozygous deleterious variant in PCDH19 (Thr146Arg) (pathogenic) was identified in the female patient EPBL-0091. The patient presented with focal epilepsy and learning disability. Pathogenic monoallelic variants in this gene are reported to exclusively affect females, a peculiar phenomenon that is explained by cellular interference in heterozygosity [12]. However, asymptomatic female carriers have been reported [13,14,15] indicating incomplete penetrance. In line with this, three of the asymptomatic mother’s sisters suffered seizures, supporting a hypothesis of maternal inheritance with broad phenotypic outcome [12].

EPBL-0094 presented with generalized epilepsy and learning disability and was found to have a heterozygous de novo splice site variant in SCN1A (c.2383-2A>C) (pathogenic). We also found a deleterious variant in a non-epilepsy gene that will be discussed in the next section.

EPBL-0097 presented with epileptic encephalopathy, GDD, and developmental motor delay. The female patient harbors a de novo frameshift variant in CDKL5 (Ser738PhefsTer46) (likely pathogenic), which causes an X-linked dominant disorder [16].

EPBL-0104 presented with generalized epilepsy. A monoallelic deleterious variant in RELN (Phe726Ser) (VUS) was found, inherited from a non-epileptic father. Di Donato et al. (2022) [17] reported incomplete penetrance and a variable phenotype for monoallelic RELN variants. The father, though epilepsy-free, suffers chronic sleep disturbances.

A de novo duplication on 3q26.32 (VUS) in patient EPBL-0107 was detected. The patient has focal epilepsy and delayed speech. Duplications in this region are associated with 3q29 microduplication syndrome, with a heterogeneous phenotype including language delay and epilepsy [18].

Patient EPBL-0145 from a consanguineous family was found to have a homozygous deleterious variant in CACNB4 (Ile378Met) (VUS). The patient presented with focal epilepsy and an undetermined psychotic disorder. The mutation is predicted by DynaMut2 to eliminate several hydrophobic bonds, destabilizing the protein with ΔΔG^Stability^ = −0.47 kcal/mol (Figure 2).

A de novo deleterious variant in SARS1 (Pro226His) (VUS) was found in patient EPBL-0165 with focal epilepsy. Deleterious variants in aminoacyl-tRNA synthetases (in this case serine) cause a spectrum of ailments depending on the variant [19], including seizures.

EPBL-0215 presented with focal epilepsy as well as intellectual and learning disabilities. A novel heterozygous CACNA1C (Asp2213Gly) (VUS) deleterious variant was found, inherited from the epileptic mother. Deleterious variants affecting this gene present with a heterogeneous phenotype [20], which may explain the lack of ID in the mother.

Finally, EPBL-0221 presented with generalized epilepsy, developmental delay and learning disability. The mother is also epileptic without comorbidities. A maternally inherited novel heterozygous deleterious variant in SPTAN1 (Lys981Arg) (VUS) was found. Deleterious variants in this gene have a heterogeneous phenotype [21], which could explain the different phenotype between child and mother.

### 2.3. Identification of Deleterious Variants in Low Confidence Genes

We also considered damaging variants in genes that are labeled amber or red on the Genomics England panel. We have found damaging variants in CACNA1H, CSNK2A1, and RYR3 as discussed below.

EPBL-0031, who is of a consanguineous family, presented with epilepsy and ASD and is diagnosed with Landau–Kleffner syndrome. A genetic test for autoimmune encephalitis in a commercial lab was negative, as was a biotinidase deficiency test. We found a heterozygous deleterious variant in RYR3 (Asp3243Tyr) (VUS) of unknown inheritance as the father was unavailable for testing. The gene has weak evidence linking it to epilepsy [22,23], but no evidence of links to Landau–Kleffner syndrome. We suspect that other factors unknown to us may be contributing to the disease.

RYR3 was affected in another patient, EPBL-0052, a female from a consanguineous family who presented with infantile spasms and epileptic encephalopathy. The RYR3 (Ala685Val) (VUS) homozygous deleterious variant is novel.

EPBL-0082 is a male with focal epilepsy. We found a novel heterozygous deleterious variant in CSNK2A1 (Arg172Leu) (likely pathogenic) with unknown origin, as the mother’s sample was unavailable. A defective CSNK2A1 is known to cause Okur–Chung neurodevelopmental syndrome (OCNDS) with autosomal dominant inheritance [24]. Although this variant lies close to a previously reported OCNDS deleterious variant (Ile174Met), our patient does not display any of the syndrome’s characteristics, apart from seizures. The discrepancy in presentation remains unexplained at this time.

In addition to variants in genes listed on the Genomics England panel, we have found damaging variants in genes previously described to cause epilepsy, among other disorders. AP4M1, described in [25,26] to cause developmental and epileptic encephalopathy, harbors a damaging homozygous variant (Tyr449Cys) (VUS) in EPBL-0019. The 9 year old male presented with ID, developmental delay and focal epilepsy.

SCYL2, whose product is involved in the agenesis of Corpus Callosum causing Arthrogryposis Multiplex Congenita [27], was found to be affected by a novel homozygous deleterious variant (Leu574Pro) (VUS) in one of our patients, EPBL-0189. The full case report can be found in [28]. The child of a third degree related parents, who presented focal epilepsy, GDD, hypotonia, quadriplegic cerebral palsy, swallowing/feeding difficulties, optic atrophy, clubfoot, and an absent corpus callosum, died at 27 months of age. Another homozygous deleterious variant was found in STAB1 (Pro1978Ser) (VUS). Some symptoms of a defective STAB1 gene overlap with those of a Arthrogryposis Multiplex Congenita, namely neurodevelopmental disorders [29,30].

An 8 year old male patient, EPBL-0193, presented with focal epilepsy and GDD and diagnosed with Angelman syndrome, had a loss of maternal allele in 15q11-q13. An extensive review of this genetic disorder can be found at [31].

We have listed other damaging variants in genes related to neurologic diseases and those which may have contributed to pathogenesis in Supplementary Table S1. Deleterious variants that have been independently verified are underlined in the table.

### 2.4. HLA-DRB Is Associated with Epilepsy

To probe the involvement of the immune system in pathogenesis, we sought to reveal associations with HLA alleles. We performed in silico typing of 13 HLA genes for all patients (Supplementary Table S1, HLA typing sheet) using HLA*LA software v1.0 [32]. HLA*LA generates allelotypes at G group level with accuracy on par with clinically certified typing methods. Challenges in WES typing led to some missing or low-confidence calls, ultimately achieving high-confidence typing for 92% of loci (excluding HLA-DRB3/4). We corroborated the findings using an alternative tool, HLA-HD [33], which employs a distinct typing method with high accuracy. As controls, we used around 2100 non-epilepsy WGS samples from the Qatar Genome Project. The total number of allelotypes found for the 13 genes in both patients and controls was 362. We performed association analysis on unrelated cases only, adjusting for sex, population structure, and multiple testing, as described in the methods. HLA types can be found in Supplementary Table S1, HLA typing sheet. DRB1*07:01:01G and DRB4*01:01:01G are associated with disease status with an odds ratio of about 3 (Table 1). DRB1*07 has been suggested as a risk allele for juvenile myoclonic epilepsy [8], and both DRB4 and DRB1*07 have been associated with anti-LGI1 encephalitis [34,35,36]. On the other hand, DRB3*01:01:02G is negatively associated with disease, with an OR of 0.5, suggesting that it may have a protective role. None of the three alleles are in linkage disequilibrium in our population.

## 3. Discussion

Epilepsy is a heterogeneous disease with varying contributions from genetic and environmental factors. As such, the list of epilepsy associated genes is constantly revised. Using a cohort from understudied and highly consanguineous populations of the MENA region, enabled us to discover novel variants in known disease genes. Analyzing the exomes of 67 epilepsy families with 81 affected members, we found monogenic variants in almost 30% of the families in 15 established epilepsy genes and 5 low confidence genes. The variants include exon and splice site SNVs, small indels, CNVs, and LOH. Thirteen SNVs were novel and six were homozygous. We also revealed damaging variants in genes not previously associated with epilepsy, but rather with neurologic pathways or disorders. Given the high consanguinity, these variants were predominantly homozygous. To better understand their potential impact, we grouped the affected genes into five mechanistic categories: (1) ion channel dysfunction, including genes such as *SCN1A*, *SCN9A*, and *CACNA1C*, which are associated with neuronal excitability; (2) synaptic transmission and vesicle trafficking, involving genes like *PRRT2* and *PCDH19*; (3) transcriptional and epigenetic regulation, such as *CDKL5* and *HECW2*, linked to early epileptic encephalopathies; (4) neuronal migration and structural integrity, represented by *RELN* and *SPTAN1*; and (5) metabolic and mitochondrial function, including *HSD17B4* and *SARS1*, whose disruption may indirectly contribute to seizure pathogenesis. This functional classification underscores the biological diversity underlying epilepsy in this cohort and may help guide future therapeutic approaches. A comprehensive list of variants, alongside supporting evidence from the literature (Supplementary Table S1), is provided for the research community’s consideration. Definitive confirmation of variant pathogenesis awaits functional studies.

The established involvement of the immune system in epilepsy is underscored by evidence pointing to both an immune response triggered by epileptic activity and its implication in epileptogenesis [37]. Specifically, HLA loci have been associated with autoimmune epilepsy (reviewed in [35]), with limited studies linking them to generalized epilepsies. The considerable heterogeneity of HLA genotypes poses challenges for disease-focused investigations, often constrained by small sample sizes and a narrow focus on selected loci. Notably, our study, the first to concurrently assess alleles of 13 HLA genes in the epilepsy context, yielded highly significant results despite a modest sample size. Our findings propose potential risk (DRB1*07:01:01G and DRB4*01:01:01G) and protective (DRB3*01:01:02G) HLA alleles. These G groups comprise alleles sharing identical nucleotide sequences across exon 2, encoding the peptide binding domains of class II genes. *HLA-DR*, a crucial cell surface molecule expressed on antigen-presenting cells, initiates the inflammatory cascade through antigen presentation to T cells. Studies in temporal lobe epilepsy (TLE) patients’ brain tissue revealed increased *HLA-DR* immunoreactive microglia, supporting inflammation’s role in epileptogenesis [38,39]. DRB1*07:01 in particular has been suggested as a risk for juvenile myoclonic epilepsy [8], and both *DRB4* and DRB1*07 have been associated with anti-LGI1 encephalitis [34,35,36,40]. A possible explanation for the risk-modifying effect of certain HLA alleles in epilepsy lies in their involvement in regulating gut microbiome composition. Previous studies have suggested that certain HLA alleles are associated with specific gut microbiota, influencing immune tolerance or promoting inflammation [41,42,43,44]. Persistent inflammation can compromise the integrity of the blood–brain barrier, allowing peripheral immune mediators to access the brain and potentially contribute to epileptogenesis.

It is important to acknowledge that our findings may not be fully generalizable across all populations, as HLA allele frequencies and their associated risk contributions can vary significantly by ancestry. For example, the DRB1*07:01 allele occurs at a frequency of 0.2 in the Qatari population, compared to 0.13 in Caucasian populations [45] reflecting ancestry-specific genetic variation. Additionally, the broader genetic background within each ethnic group may modulate the risk conferred by individual HLA alleles. These population-level differences, along with variation in HLA typing methodologies and disease characteristics, likely contribute to the lack of replication for some alleles previously associated with non-autoimmune forms of epilepsy in our study.

GWAS’s have proven instrumental in elucidating the involvement of HLA in numerous diseases; but with limited success in epilepsy. A recent GWAS meta-analysis, predominantly focused on individuals of Caucasian ancestry, failed to identify a significant genome-wide signal within the HLA loci [46]. Given the limited statistical power of individual SNP signals, we propose a gene- or exon-based analytical approach, which necessitates the use of specialized bioinformatics tools for this region.

The identification of risk or protective alleles in epilepsy patients holds potential for elucidating the phenotypic variability observed in established genetic epilepsies. Understanding the influence of specific alleles could contribute valuable insights into the diverse clinical manifestations associated with these well-known genetic epilepsies.

This study emphasizes the need for genetic testing, particularly in consanguineous populations, to help reduce the burden of disease through proper genetic counseling and informed reproductive choices. Furthermore, knowledge of the genetic profile has led clinicians to tailor anticonvulsant therapy, avoid contraindicated drugs, or initiate metabolic therapies such as ketogenic diets.

## 4. Materials and Methods

### 4.1. Sample Collection and Processing

Participants and/or guardians provided informed consent prior to participation in the study in accordance with the Declaration of Helsinki and the Institutional Review Board approved protocol (protocol number #1708013656). Blood (2.5 mL/kg of body weight) was drawn (maximum of 5 mL for infants and 50 mL for adult subjects) from each subject into sodium heparin tubes, and PBMCs were isolated from blood by ficoll-gradient separation. DNA was isolated from PBMCs using the DNeasy Blood and Tissue Kit (Qiagen, Hilden, Germany), following the manufacturer’s recommendations. DNA quantity and quality were checked using a Nanodrop spectrophotometer (ThermoScientific, Waltham, MA, USA) and sent for whole-exome analysis (WES) at Sidra Genomics Core facility. Agilent (Santa Clara, CA, USA) SureSelect XT low input kit V6 probe set was used for exome enrichment. DNA processed for WES was subjected to Illumina (San Diego, CA, USA) HiSeq sequencing, generating 150 bp paired end reads with an average of 70 million reads and >50× coverage.

### 4.2. Genetic Data Analysis

Quality control was performed for gender and relatedness using PLINK 1.9 [47]. The reads were aligned with a bowtie [48] to GRCh37 reference genome. More than 99% of the reads aligned. SNPs and indels were called with GATK [49]; CNVs were detected with NxClinical software v5.1 (Bionano Genomics, San Diego, CA, USA) a package that detects CNVs and LOHs from BAM files based on read depth and a pooled reference, integrates up-to-date several public databases and variant annotation tools, and allows for filtering of variants resulting from the uploaded BAM and VCF files. We applied the following filters for SNPs and indels: read depth > 20, MAPQ  >  30, base quality > 20, MAF  <  0.01 (1%), in silico prediction of pathogenicity by SIFT [50] and/or PolyPhen [51]. CNVs with overlapping segments in the Database of Genomic Variants (DGV) [52] or those that are common in our internal database of Middle Eastern subjects were excluded. Further filtering for known epilepsy genes was performed by uploading the Genomics England epilepsy panel gene list to NxClinical. Candidate gene variants were selected based on a scoring system implemented by NxClinical, assessing the relevance of the affected gene to the phenotype based on textual associations of genes with epilepsy in the scientific literature. Initially, we looked for all homozygous damaging variants and re-examined the affected genes for relevance with epilepsy and the variants for familial co-segregation with disease status. In a few exceptions, where incomplete penetrance was suspected, co-segregation was overlooked. The ACMG interpretation of variants was based on VarSome Premium (13 July 2022; updated on 20 May 2025), an online tool that integrates several up-to-date variant prediction and annotation databases (around 140). Since VarSome does not take into consideration variant co-segregation with disease in the family, an important ACMG guideline for variant classification, we kept variants of uncertain significance (VUS) in addition to pathogenic and likely pathogenic variants (P/LP). Pedigrees were drawn with Kinship2 v1.8.5 [53]. Protein structures and pathogenicity scores were generated in AlphaMissense (accessed via https://alphafold.ebi.ac.uk/ on 1 May 2025) [54]. The predicted structures were then uploaded to DynaMut2 (accessed via https://biosig.lab.uq.edu.au/dynamut2/ on 1 May 2025) [55], which generated cartoon representations for the wild type and mutant proteins together with the predicted ΔΔG^Stability^ scores.

### 4.3. HLA Typing and Analysis

HLA typing was performed in silico using HLA*LA software [32]. Reads mapping to the MHC region and unmapped reads were extracted and used to type genes *A*, *B*, *C*, *DPA1*, *DPB1*, *DQA1*, *DQB1*, *DRB1*, *DRB3*, *DRB4*, *E*, *F*, and *G* to group level. Alleles were considered missing if they had coverage below 10 reads, a proportionKmersCovered value of −1, or lacked a corresponding perfect G group match. HLA-HD [56], another HLA typing software, was used to verify the results using default parameters. Data from probands excluding direct relatives was used to find allotypes associated with epilepsy (a total of 67 samples). A total of 2107 control samples of non-related, non-epilepsy WGS from the Qatar Genome Program [57] were typed similarly with HLA*LA. PLINK 1.9 was used to perform principal component analysis on control and case samples (Supplementary Figure S1). Perl and Shell scripts were used to prepare input files for PLINK to perform a logistic regression analysis with gender and the first 4 PCs as covariates. *p* values were corrected for multiple testing of all alleles with the Bonferroni method. Linkage disequilibrium analysis was performed with PLINK and a cut off r^2^ > 0.7.

## 5. Conclusions

The genetic investigation of a cohort of patients from the MENA region revealed novel pathogenic variants with autosomal dominant, autosomal recessive, X-linked, and reduced penetrant inheritance. The affected genes include well-established epilepsy genes: *ALKBH8*, *ATP1A2*, *CACNA1C*, *CACNB4*, *CDKL5*, *HECW2*, *HSD17B4*, *KCTD3*, *PCDH19*, *PRRT2*, *RELN*, *RNASEH2B*, *SARS1*, *SCN1A*, *SPTAN1*, and less established genes: *CACNA1H*, *CSNK2A1*, *RYR3*, and *SCN9A*. Functional studies are required to confirm the causality between the reported variants and epilepsy. Genetic susceptibility was found in HLA class II genes, where group alleles DRB1*07:01:01G and DRB4*01:01:01G were positively associated with disease, and DRB3*01:01:02G had a negative association. The replication of these associations warrants larger cohorts for robust validation.

## Figures and Tables

**Table 1:** HLA allotypes associated with epilepsy status. The table shows the frequencies of alleles in controls and cases in the respective locus and their counts, the odds ratio (OR) and 95% confidence interval, *p* adjusted to all tests using Bonferroni method, and the predicted role in pathogenesis.

Allelotype	Freq (Count) Controls	Freq (Count) Cases	OR	Confidence Interval	*p* Adj
DRB1*07:01:01G	0.20 (622)	0.31 (34)	3.2	0.6–5.7	4.0 × 10^−6^
DRB3*01:01:02G	0.49 (2054)	0.35 (26)	0.5	−1.8–2.8	2.0 × 10^−5^
DRB4*01:01:01G	0.93 (1328)	1.00 (75)	3.2	0.9–5.6	5.1 × 10^−9^

## Data Availability

The authors confirm that the data supporting the findings of this study are available within the article and its additional material. WES data cannot be shared because it contains information that could compromise the privacy of research participants.

## Supplementary Materials

The supporting information can be downloaded at: https://www.mdpi.com/article/10.3390/ijms26125815/s1.

## Abbreviations

The following abbreviations are used in this manuscript:

ACMG	American College of Medical Genetics and Genomics
ASD	Autism Spectrum Disorder
CNV	Copy Number Variant
DGV	Database of Genomic Variants
EEG	Electroencephalogram
GATK	Genome Analysis Toolkit
GDD	Global Developmental Delay
GWAS	Genome-Wide Association Studies
HLA	Human Leukocyte Antigen
ID	Intellectual Disability
ILAE	International League Against Epilepsy
LOH	Loss of Heterozygosity
MAF	Minor Allele Frequency
MAPQ	Mapping Quality
MENA	Middle East and North Africa
OCNDS	Okur–Chung Neurodevelopmental Syndrome
OMIM	Online Mendelian Inheritance in Man
P/LP	Pathogenic / Likely Pathogenic
PBMC	Peripheral Blood Mononuclear Cell
SNP	Single Nucleotide Polymorphism
VUS	Variant of Uncertain Significance
WES	Whole Exome Sequencing
WGS	Whole Genome Sequencing

## References

[1] Dunn P., Albury C.L., Maksemous N., Benton M.C., Sutherland H.G., Smith R.A., Haupt L.M., Griffiths L.R. (2018). Next Generation Sequencing Methods for Diagnosis of Epilepsy Syndromes. Front. Genet..

[2] Neligan A., Hauser W.A., Sander J.W. (2012). The Epidemiology of the Epilepsies. Handbook of Clinical Neurology.

[3] Sánchez Fernández I., Loddenkemper T., Gaínza-Lein M., Sheidley B.R., Poduri A. (2019). Diagnostic Yield of Genetic Tests in Epilepsy: A Meta-Analysis and Cost-Effectiveness Study. Neurology.

[4] Langenbruch L., Bleß L., Schulte-Mecklenbeck A., Sundermann B., Brix T., Elger C.E., Melzer N., Wiendl H., Meuth S.G., Gross C.C. (2020). Blood and Cerebrospinal Fluid Immune Cell Profiles in Patients with Temporal Lobe Epilepsy of Different Etiologies. Epilepsia.

[5] Leal B., Chaves J., Carvalho C., Rangel R., Santos A., Bettencourt A., Lopes J., Ramalheira J., Silva B.M., da Silva A.M. (2017). Brain Expression of Inflammatory Mediators in Mesial Temporal Lobe Epilepsy Patients. J. Neuroimmunol..

[6] Owens G.C., Garcia A.J., Mochizuki A.Y., Chang J.W., Reyes S.D., Salamon N., Prins R.M., Mathern G.W., Fallah A. (2019). Evidence for Innate and Adaptive Immune Responses in a Cohort of Intractable Pediatric Epilepsy Surgery Patients. Front. Immunol..

[7] Benedek G., El Latif M.A., Miller K., Rivkin M., Lasu A.A.R., Riek L.P., Lako R., Edvardson S., Alon S.-A., Galun E. (2020). Protection or Susceptibility to Devastating Childhood Epilepsy: Nodding Syndrome Associates with Immunogenetic Fingerprints in the HLA Binding Groove. PLoS Neglected Trop. Dis..

[8] Chaves J., Martins-Ferreira R., Ferreira A.M., Brás S., Carvalho C., Bettencourt A., Samões R., Monteiro F., Freitas J., Chorão R. (2020). Immunogenetic Protective Factors in Genetic Generalized Epilepsy. Epilepsy Res..

[9] Scheffer I.E., Berkovic S., Capovilla G., Connolly M.B., French J., Guilhoto L., Hirsch E., Jain S., Mathern G.W., Moshé S.L. (2017). ILAE Classification of the Epilepsies: Position Paper of the ILAE Commission for Classification and Terminology. Epilepsia.

[10] Martin A.R., Williams E., Foulger R.E., Leigh S., Daugherty L.C., Niblock O., Leong I.U.S., Smith K.R., Gerasimenko O., Haraldsdottir E. (2019). PanelApp Crowdsources Expert Knowledge to Establish Consensus Diagnostic Gene Panels. Nat. Genet..

[11] Ghanty I., Perez-Palma E., Villaman C., Stobo D., Symonds J., Zuberi S., Lal D., Brunklaus A. (2025). *SCN9A* Should Not Be Considered an Epilepsy Gene; Refuting a Gene–Disease Association. Epilepsia.

[12] Dell’Isola G.B., Vinti V., Fattorusso A., Tascini G., Mencaroni E., Di Cara G., Striano P., Verrotti A. (2022). The Broad Clinical Spectrum of Epilepsies Associated with Protocadherin 19 Gene Mutation. Front. Neurol..

[13] Dimova P.S., Kirov A., Todorova A., Todorov T., Mitev V. (2012). A Novel PCDH19 Mutation Inherited from an Unaffected Mother. Pediatr. Neurol..

[14] Hung L.-Y., Subramaniam S.R., Tong T.-Y.T., Chan W.-K., Yau E.K.-C., Ching C.-K. (2021). X-Chromosome Inactivation and PCDH19-Associated Epileptic Encephalopathy: A Novel PCDH19 Variant in a Chinese Family. Clin. Chim. Acta.

[15] Ryan S.G., Chance P.F., Zou C.-H., Spinner N.B., Golden J.A., Smietana S. (1997). Epilepsy and Mental Retardation Limited to Females: An X-Linked Dominant Disorder with Male Sparing. Nat. Genet..

[16] Weaving L.S., Christodoulou J., Williamson S.L., Friend K.L., McKenzie O.L.D., Archer H., Evans J., Clarke A., Pelka G.J., Tam P.P.L. (2004). Mutations of CDKL5 Cause a Severe Neurodevelopmental Disorder with Infantile Spasms and Mental Retardation. Am. J. Hum. Genet..

[17] Di Donato N., Guerrini R., Billington C.J., Barkovich A.J., Dinkel P., Freri E., Heide M., Gershon E.S., Gertler T.S., Hopkin R.J. (2022). Monoallelic and Biallelic Mutations in *RELN* Underlie a Graded Series of Neurodevelopmental Disorders. Brain.

[18] Coyan A.G., Dyer L.M. (2020). 3q29 Microduplication Syndrome: Clinical and Molecular Description of Eleven New Cases. Eur. J. Med. Genet..

[19] Bögershausen N., Krawczyk H.E., Jamra R.A., Lin S., Yigit G., Hüning I., Polo A.M., Vona B., Huang K., Schmidt J. (2022). *WARS1* and *SARS1*: Two tRNA Synthetases Implicated in Autosomal Recessive Microcephaly. Hum. Mutat..

[20] Bozarth X., Dines J.N., Cong Q., Mirzaa G.M., Foss K., Lawrence Merritt J., Thies J., Mefford H.C., Novotny E. (2018). Expanding Clinical Phenotype in *CACNA1C* Related Disorders: From Neonatal Onset Severe Epileptic Encephalopathy to Late-Onset Epilepsy. Am. J. Med. Genet. A..

[21] Marco Hernández A.V., Caro A., Montoya Filardi A., Tomás Vila M., Monfort S., Beseler Soto B., Nieto-Barceló J.J., Martínez F. (2022). Extending the Clinical Phenotype of *SPTAN1*: From DEE5 to Migraine, Epilepsy, and Subependymal Heterotopias Without Intellectual Disability. Am. J. Med. Genet. A.

[22] Appenzeller S., Balling R., Barisic N., Baulac S., Caglayan H., Craiu D., De Jonghe P., Depienne C., Dimova P., Djémié T. (2014). De Novo Mutations in Synaptic Transmission Genes Including DNM1 Cause Epileptic Encephalopathies. Am. J. Hum. Genet..

[23] Peng J., Wang Y., He F., Chen C., Wu L., Yang L., Ma Y., Zhang W., Shi Z., Chen C. (2018). Novel West Syndrome Candidate Genes in a Chinese Cohort. CNS Neurosci. Ther..

[24] Jafari Khamirani H., Zoghi S., Motealleh A., Dianatpour M., Tabei S.M.B., Mohammadi S., Dastgheib S.A. (2022). Clinical Features of Okur-Chung Neurodevelopmental Syndrome: Case Report and Literature Review. Mol. Syndromol..

[25] Oliver K.L., Scheffer I.E., Bennett M.F., Grinton B.E., Bahlo M., Berkovic S.F. (2023). Genes4Epilepsy: An Epilepsy Gene Resource. Epilepsia.

[26] Becker A., Felici C., Lambert L., De Saint Martin A., Abi-Warde M., Schaefer E., Zix C., Zamani M., Sadeghian S., Zeighami J. (2023). Putative Founder Effect of Arg338* *AP4M1* (SPG50) Variant Causing Severe Intellectual Disability, Epilepsy and Spastic Paraplegia: Report of Three Families. Clin. Genet..

[27] Seidahmed M.Z., Al-Kindi A., Alsaif H.S., Miqdad A., Alabbad N., Alfifi A., Abdelbasit O.B., Alhussein K., Alsamadi A., Ibrahim N. (2020). Recessive Mutations in SCYL2 Cause a Novel Syndromic Form of Arthrogryposis in Humans. Hum. Genet..

[28] Zamel K., Al-Subaiey A.A., Alsabbagh M., Fadda A., Saeed A., Mourao Pacheco B., Lo B., Benini R. (2025). Novel SCYL2 Mutations and Arthrogryposis Multiplex Congenita 4: Case Report and Review of the Literature. Int. J. Mol. Sci..

[29] den Hoed J., de Boer E., Voisin N., Dingemans A.J.M., Guex N., Wiel L., Nellaker C., Amudhavalli S.M., Banka S., Bena F.S. (2021). Mutation-Specific Pathophysiological Mechanisms Define Different Neurodevelopmental Disorders Associated with SATB1 Dysfunction. Am. J. Hum. Genet..

[30] Yu Y., Li C., Li W., Chen L., Wang D., Wang J., Wang J., Yao R. (2022). Neurodevelopmental Disorders and Anti-Epileptic Treatment in a Patient with a SATB1 Mutation: A Case Report. Front. Pediatr..

[31] Butler M.G., Duis J. (2020). Chromosome 15 Imprinting Disorders: Genetic Laboratory Methodology and Approaches. Front. Pediatr..

[32] Dilthey A.T., Mentzer A.J., Carapito R., Cutland C., Cereb N., Madhi S.A., Rhie A., Koren S., Bahram S., McVean G. (2019). HLA*LA-HLA Typing from Linearly Projected Graph Alignments. Bioinformatics.

[33] Kawaguchi S., Higasa K., Shimizu M., Yamada R., Matsuda F. (2017). HLA-HD: An Accurate HLA Typing Algorithm for Next-Generation Sequencing Data. Hum. Mutat..

[34] Kim T.-J., Lee S.-T., Moon J., Sunwoo J.-S., Byun J.-I., Lim J.-A., Shin Y.-W., Jun J.-S., Lee H.S., Lee W.-J. (2017). Anti-LGI1 Encephalitis Is Associated with Unique HLA Subtypes: HLA Subtypes in Anti-LGI1 Encephalitis. Ann. Neurol..

[35] Muñiz-Castrillo S., Vogrig A., Honnorat J. (2020). Associations between HLA and Autoimmune Neurological Diseases with Autoantibodies. Auto. Immun. Highlights.

[36] van Sonderen A., Roelen D.L., Stoop J.A., Verdijk R.M., Haasnoot G.W., Thijs R.D., Wirtz P.W., Schreurs M.W.J., Claas F.H.J., Sillevis Smitt P.A.E. (2017). Anti-LGI1 Encephalitis Is Strongly Associated with HLA-DR7 and HLA-DRB4. Ann. Neurol..

[37] Vezzani A., French J., Bartfai T., Baram T.Z. (2011). The Role of Inflammation in Epilepsy. Nat. Rev. Neurol..

[38] Leal B., Chaves J., Carvalho C., Bettencourt A., Brito C., Boleixa D., Freitas J., Brás S., Lopes J., Ramalheira J. (2018). Immunogenetic Predisposing Factors for Mesial Temporal Lobe Epilepsy with Hippocampal Sclerosis. Int. J. Neurosci..

[39] Peixoto-Santos J.E., Kandratavicius L., Velasco T.R., Assirati J.A., Carlotti C.G., Scandiuzzi R.C., Salmon C.E.G., Santos A.C.D., Leite J.P. (2017). Individual Hippocampal Subfield Assessment Indicates That Matrix Macromolecules and Gliosis Are Key Elements for the Increased T2 Relaxation Time Seen in Temporal Lobe Epilepsy. Epilepsia.

[40] Peris Sempere V., Muñiz-Castrillo S., Ambati A., Binks S., Pinto A.-L., Rogemond V., Pittock S.J., Dubey D., Geschwind M.D., Gelfand J.M. (2022). Human Leukocyte Antigen Association Study Reveals DRB1*04:02 Effects Additional to DRB1*07:01 in Anti-LGI1 Encephalitis. Neurol. Neuroimmunol. Neuroinflamm..

[41] Andeweg S.P., Keşmir C., Dutilh B.E. (2021). Quantifying the Impact of Human Leukocyte Antigen on the Human Gut Microbiota. mSphere.

[42] Shahi S.K., Ali S., Jaime C.M., Guseva N.V., Mangalam A.K. (2021). HLA Class II Polymorphisms Modulate Gut Microbiota and Experimental Autoimmune Encephalomyelitis Phenotype. ImmunoHorizons.

[43] Parker A., Fonseca S., Carding S.R. (2020). Gut Microbes and Metabolites as Modulators of Blood-Brain Barrier Integrity and Brain Health. Gut Microbes.

[44] Russell J.T., Roesch L.F.W., Ördberg M., Ilonen J., Atkinson M.A., Schatz D.A., Triplett E.W., Ludvigsson J. (2019). Genetic Risk for Autoimmunity Is Associated with Distinct Changes in the Human Gut Microbiome. Nat. Commun..

[45] Gonzalez-Galarza F.F., McCabe A., Santos E.J.M.D., Jones J., Takeshita L., Ortega-Rivera N.D., Cid-Pavon G.M.D., Ramsbottom K., Ghattaoraya G., Alfirevic A. (2020). Allele Frequency Net Database (AFND) 2020 Update: Gold-Standard Data Classification, Open Access Genotype Data and New Query Tools. Nucleic. Acids Res..

[46] Stevelink R., Campbell C., Chen S., Abou-Khalil B., Adesoji O.M., Afawi Z., Amadori E., Anderson A., Anderson J., International League Against Epilepsy Consortium on Complex Epilepsies (2023). GWAS Meta-Analysis of over 29,000 People with Epilepsy Identifies 26 Risk Loci and Subtype-Specific Genetic Architecture. Nat. Genet..

[47] Purcell S., Neale B., Todd-Brown K., Thomas L., Ferreira M.A.R., Bender D., Maller J., Sklar P., de Bakker P.I.W., Daly M.J. (2007). PLINK: A Tool Set for Whole-Genome Association and Population-Based Linkage Analyses. Am. J. Hum. Genet..

[48] Langmead B., Trapnell C., Pop M., Salzberg S.L. (2009). Ultrafast and Memory-Efficient Alignment of Short DNA Sequences to the Human Genome. Genome. Biol..

[49] McKenna A., Hanna M., Banks E., Sivachenko A., Cibulskis K., Kernytsky A., Garimella K., Altshuler D., Gabriel S., Daly M. (2010). The Genome Analysis Toolkit: A MapReduce Framework for Analyzing Next-Generation DNA Sequencing Data. Genome. Res..

[50] Kumar P., Henikoff S., Ng P.C. (2009). Predicting the Effects of Coding Non-Synonymous Variants on Protein Function Using the SIFT Algorithm. Nat. Protoc..

[51] Adzhubei I.A., Schmidt S., Peshkin L., Ramensky V.E., Gerasimova A., Bork P., Kondrashov A.S., Sunyaev S.R. (2010). A Method and Server for Predicting Damaging Missense Mutations. Nat. Methods.

[52] MacDonald J.R., Ziman R., Yuen R.K.C., Feuk L., Scherer S.W. (2014). The Database of Genomic Variants: A Curated Collection of Structural Variation in the Human Genome. Nucleic Acids Res..

[53] Sinnwell J.P., Therneau T.M., Schaid D.J. (2014). The Kinship2 R Package for Pedigree Data. Hum. Hered..

[54] Cheng J., Novati G., Pan J., Bycroft C., Žemgulytė A., Applebaum T., Pritzel A., Wong L.H., Zielinski M., Sargeant T. (2023). Accurate Proteome-Wide Missense Variant Effect Prediction with AlphaMissense. Science.

[55] Rodrigues C.H.M., Pires D.E.V., Ascher D.B. (2021). DynaMut2: Assessing Changes in Stability and Flexibility upon Single and Multiple Point Missense Mutations. Protein Sci..

[56] Kawaguchi S., Higasa K., Yamada R., Matsuda F., Boegel S. (2018). Comprehensive HLA Typing from a Current Allele Database Using Next-Generation Sequencing Data. HLA Typing.

[57] Mbarek H., Devadoss Gandhi G., Selvaraj S., Al-Muftah W., Badji R., Al-Sarraj Y., Saad C., Darwish D., Alvi M., Fadl T. (2022). Qatar Genome: Insights on Genomics from the Middle East. Hum. Mutat..

