# Supplementary material for: The Complex Etiology of Epilepsy: Genetic Analysis and HLA Association in Patients in the Middle East
Source: Int J Mol Sci. 2025 Jun 17;26(12):5815. doi: 10.3390/ijms26125815 (PMC12193080; doi:10.3390/ijms26125815)
Supplement: Supplementary file 1 [file ijms-26-05815-s001.zip › Figure S2.pdf]

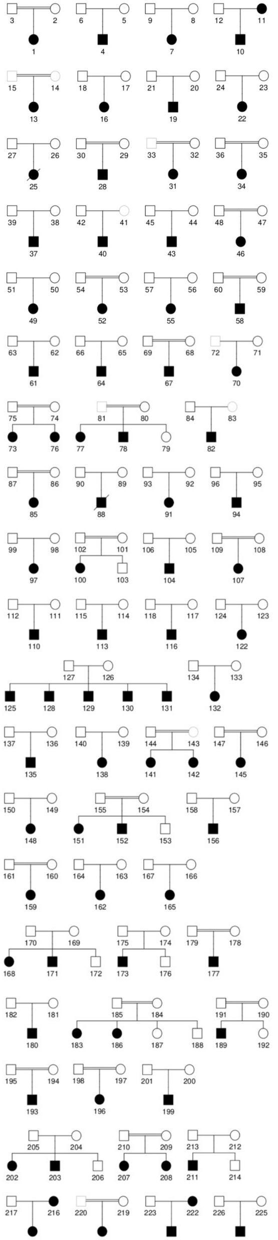
3 2 6 5 9 8 12 11
1 4 7 10
15 14 18 17 21 20 24 23
13 16 19 22
27 26 30 29 33 32 36 35
25 28 31 34
39 38 42 41 45 44 48 47
37 40 43 46
51 50 54 53 57 56 60 59
49 52 55 58
63 62 66 65 69 68 72 71
61 64 67 70
75 74 81 80 84 83
73 76 77 78 79 82
87 86 90 89 93 92 96 95
85 88 91 94
99 98 102 101 106 105 109 108
97 100 103 104 107
112 111 115 114 118 117 124 123
110 113 116 122
127 126 134 133
125 128 129 130 131 132
137 136 140 139 144 143 147 146
135 138 141 142 145
150 149 155 154 158 157
148 151 152 153 156
161 160 164 163 167 166
159 162 165
170 169 175 174 179 178
168 171 172 173 176 177
182 181 185 184 191 190
180 183 186 187 188 189 192
195 194 198 197 201 200
193 196 199
205 204 210 209 213 212
202 203 206 207 208 211 214
217 216 220 219 223 222 226 225
215 218 221 224

Figure S2. Pedigrees of probands with siblings that participated in the study. Circles: female; square: male; filled shapes: affected, light gray shape: parent refused to participate; diagonal line: deceased; double line: consanguineous
