# Supplementary figures and images for: The Complex Etiology of Epilepsy: Genetic Analysis and HLA Association in Patients in the Middle East
Source: Int J Mol Sci. 2025 Jun 17;26(12):5815. doi: 10.3390/ijms26125815 (PMC12193080; doi:10.3390/ijms26125815)

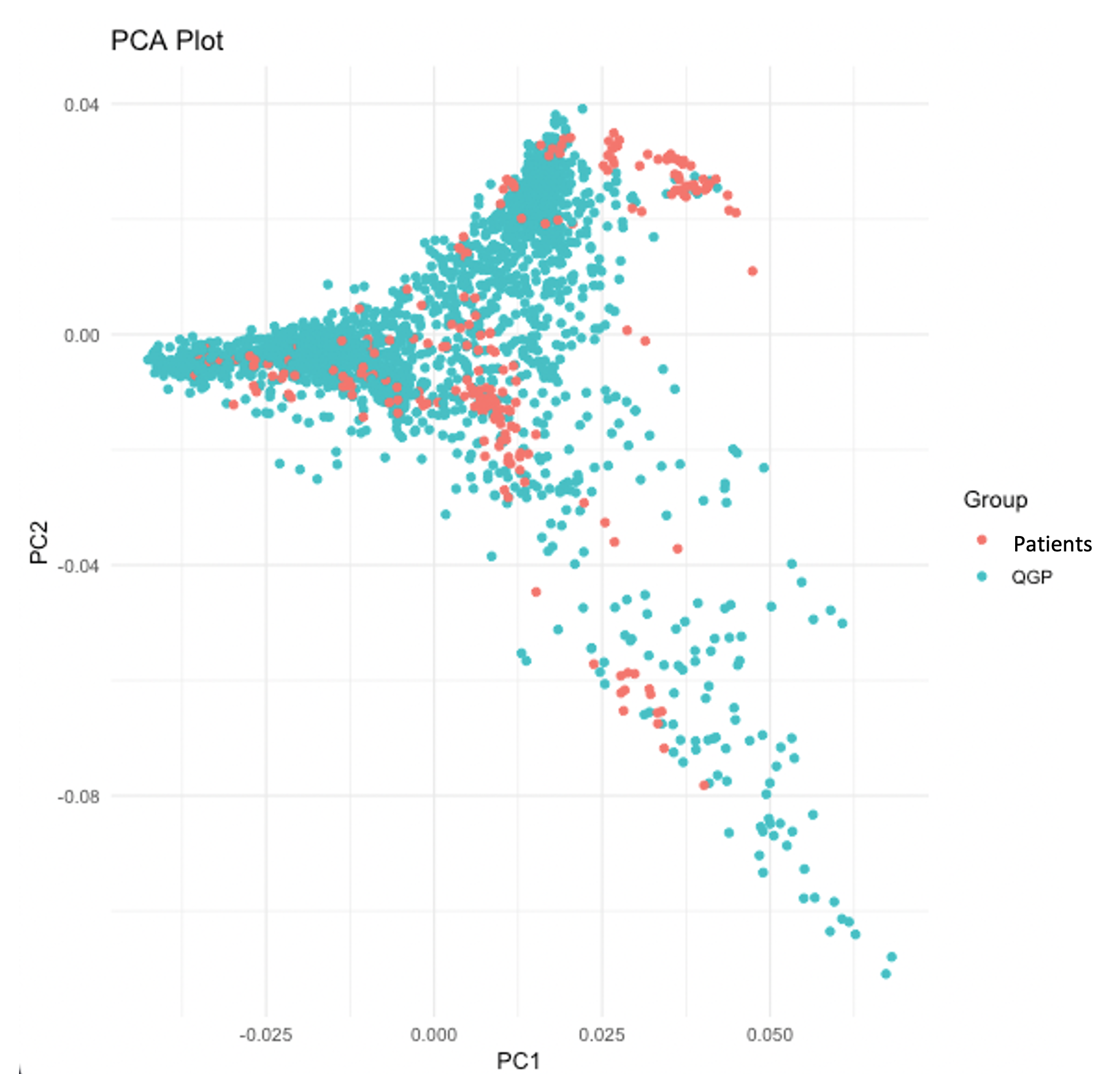

Supplement: Supplementary file 1 [file ijms-26-05815-s001.zip › Figure S1. PCA plot.png]
